# Anomalía de Jordans en síndrome de Chanarin-Dorfman

**DOI:** 10.1515/almed-2024-0073

**Published:** 2024-07-26

**Authors:** Jorge Sánchez-Cortés, Xavier Gabaldó-Barrios

**Affiliations:** Servicio de Análisis Clínicos, 16259 Hospital Universitari Sant Joan de Reus, Reus, España

**Keywords:** anomalía de Jordans, frotis de sangre periférica, síndrome de Chanarin-Dorfman

## Abstract

**Objetivos:**

El síndrome de Chanarin-Dorfman es un síndrome raro de herencia autosómica recesiva cuya prevalencia no supera los 130 casos en el mundo.

**Caso clínico:**

Paciente de 4 años afecto de síndrome ictiosiforme eritemato-descamativo generalizado desde los primeros días del nacimiento. En el informe de laboratorio destacó hipertransaminemia persistente en el tiempo. Entre otras pruebas complementarias, se realizó el frotis de sangre periférica (SP), revelando la presencia de múltiples vacuolas citoplasmáticas en el interior de los leucocitos polimorfonucleares (PMN) y plaquetas. Las lesiones ictiosiformes junto con la presencia de vacuolas lipídicas en los PMN de SP son signos compatibles con síndrome de Chanarin-Dorfman. El diagnóstico se confirmó mediante secuenciación genética.

**Conclusiones:**

El síndrome de Chanarin-Dorfman está caracterizado por una mutación del gen *CGI-58*, el cual está implicado en el catabolismo de los triglicéridos de cadena larga almacenados en gotas lipídicas citoplasmáticas. La anomalía de Jordans es un rasgo congénito caracterizado por la presencia de abundantes vacuolas en la serie granulocítica debido a defectos en el metabolismo lipídico. En este síndrome, los triglicéridos de cadena larga se depositan en los tejidos produciendo principalmente manifestaciones dermatológicas controlables mediante la restricción de los mismos en la dieta.

## Introducción

Presentamos un caso de síndrome de Chanarin-Dorfman, una afectación extremadamente rara, autosómica recesiva y multisistémica caracterizada por la incapacidad de metabolizar los lípidos neutros. Se trata de una condición muy poco frecuente, cuya prevalencia es inferior a los 130 casos mundialmente. La mayoría de los mismos se concentran en la región mediterránea, especialmente en Turquía, y se pueden relacionar con altas tasas de consanguinidad [1].

## Caso clínico

Paciente de 4 años sin antecedentes familiares de interés derivado a la consulta de dermatología por afectación de síndrome ictiosiforme congénito desde el nacimiento. Se observaron placas eritematosas con borde descamativo activo con afectación en cuero cabelludo, cara, tronco, codos, rodillas, extremidades, palmas y plantas. La biopsia cutánea reveló hiperqueratosis compatible con eccema seborreico.

Los resultados de laboratorio fueron normales salvo la aspartato aminotransferasa (AST), la alanina aminotransferasa (ALT) y la fosfatasa alcalina (FAL) (Tabla 1). Se observó hipertransaminemia persistente en el tiempo. La morfología clínica de las lesiones sugirió el diagnóstico clínico de probable eritroqueratodermia variabilis dado el carácter cambiante y migratorio de las lesiones hiperqueratósicas. Sin embargo, la alteración de las pruebas hepáticas obligó a valorar otras posibilidades. El aspecto ictiosiforme del proceso asociado a hepatopatía precisó descartar el síndrome de Chanarin Dorfman.

**Tabla 1:** Resultados analíticos de las aminotransferasas y fosfatasa alcalina.

Magnitud biológica	Resultado	Valores de referencia
AST	67,2 U/L	0–52 U/L
ALT	67,2 U/L	0–39 U/L
FAL	908,4 U/L	0–269 U/L

ALT, alanina aminotransferasa; AST, aspartato aminotransferasa; FAL, fosfatasa alcalina.

**Figura 1:**
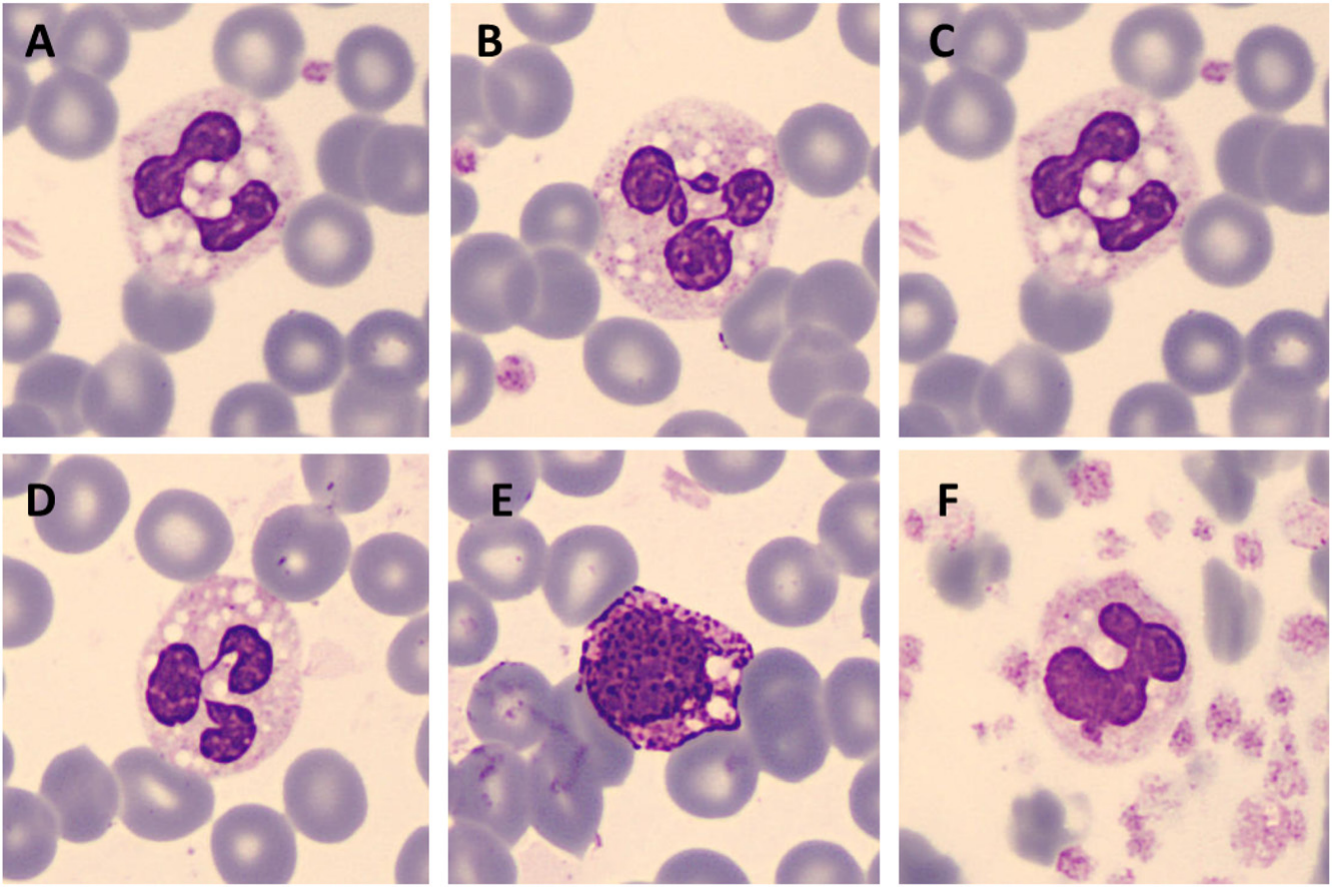
Se observa vacuolación en los neutrófilos (A–D), en los basófilos (E) y en las plaquetas (F). Imágenes obtenidas por CellaVision^®^ DM96.

Se solicitó la revisión del frotis de sangre periférica (SP), revelando múltiples vacuolas citoplasmáticas en el interior de las células de la serie granulocítica y plaquetas (Figura 1). Así, el cuadro de eritrodermia ictiosiforme seca generalizada y la presencia de vacuolas lipídicas en los polimorfonucleares (PMN) de SP sugirieron que el diagnóstico más probable era el de la enfermedad por depósito de los lípidos neutros, también conocida como síndrome de Chanarin-Dorfman. El examen del frotis de SP de los padres del paciente no mostró presencia de vacuolas en los PMN.

El diagnóstico se confirmó mediante el método de secuenciación de Sanger del gen *CGI-58*, causante de dicho síndrome. Se llevó a cabo en el caso índice y padres, detectándose una mutación sin sentido en el exón 6 (934G→T; R312X) heredada del padre y una inserción/deleción en la posición 617 en el exón 4 heredada de la madre. Esta última provocó un cambio en el marco de lectura que originó un codón de terminación prematuro. El paciente resultó ser portador de las dos mutaciones en homocigosis.

A raíz del diagnóstico, se recomendó estudio oftalmológico, valoración de las enzimas musculares, estudio otorrinolaringológico para descartar trastornos de audición y examen neuropediátrico. Adicionalmente, se aconsejó realizar controles periódicos por el servicio de dermatología y se contactó con el servicio de nutrición y dietética para hacer una valoración. La exploración por oftalmología y neuropediatría fue anodina; sin embargo, sí se objetivó hipoacusia neurosensorial leve y niveles de creatina cinasa de 2 a 5 veces el límite superior de normalidad a pesar de no presentar dolores musculares.

Dado que no existe tratamiento específico para esta enfermedad, el único tratamiento eficaz fue la restricción de los triglicéridos de cadena larga de la dieta y la suplementación con triglicéridos de cadena media. Tras seis meses de tratamiento, el paciente presentó mejoría clínica manifestando una reducción de las áreas descamativas, sin encontrar una variación significativa en los niveles de AST, ALT y FAL.

## Discusión

Jordans fue pionero en el descubrimiento de vacuolas apolares abundantes y de diversos tamaños en los leucocitos de un frotis de SP en dos hermanos afectos de distrofia muscular progresiva. Tras realizar una tinción vital con el método de Casaris-Demel (una mezcla de azul de cresilo brillante, Sudan III y alcohol absoluto), observó dichas inclusiones de color rojo en el citoplasma de la mayoría de los neutrófilos, eosinófilos y basófilos, hecho que confirmó su naturaleza lipídica. No se detectó glucógeno en dichas vacuolas [2]. Por ello, hoy en día, se le conoce a este rasgo como anomalía de Jordans. En 1974, Dorfman y cols. describieron el caso de un paciente varón de 35 años con escamas blancas en la piel junto con la ya descrita anomalía de Jordans. Un año después, Chanarin y cols. explicaron otro caso con los mismos rasgos en una mujer de 22 años. La acumulación de lípidos en el hígado y la ausencia de esta anomalía en sus familiares fueron signos suficientes para sospechar de un síndrome sistémico caracterizado por un defecto en el metabolismo de los lípidos [3], 4].

Chanarin-Dorfman es un síndrome raro caracterizado por ictiosis en la piel y la presencia de vacuolas lipídicas citoplasmáticas abundantes en varios tejidos, predominando en la serie granulocítica de la SP, en la piel y en el hígado [5]. Se asocia con una multitud de síntomas clínicos de los cuales la eritrodermia ictiosiforme congénita y esteatosis hepática son comunes a todos los pacientes. La afectación de otros órganos y sistemas como los ojos, oídos y músculos es mucho más variable. Algunos de estos síntomas son ectropión bilateral, cataratas, estrabismo, nistagmo, alopecia, microtia (orejas pequeñas) con sordera neurosensorial bilateral, hepatoesplenomegalia, y en algunos casos menos frecuentes, retraso mental y baja estatura. Dichos síntomas tienden a variar según el origen étnico de los pacientes y las diferentes mutaciones que presentan [1], 5]. La presencia de anomalía de Jordans en varios tejidos y la ictiosis comportan los rasgos clínicos comúnmente empleados en el diagnóstico de Chanarin-Dorfman.

Aunque las gotas lipídicas de este síndrome se observan mayormente en el citoplasma de las células sanguíneas, también pueden observarse en biopsias de la piel, la médula ósea y el hígado, y en menor medida puede encontrarse en el intestino delgado, el estómago y el riñón [6], [7], [8]. Además, en los escasos casos en los que la anomalía de Jordans no se manifiesta en las células de sangre periférica, se puede apreciar el acúmulo de vacuolas lipídicas en biopsias de piel mediante microscopía electrónica de transición [9].

Las gotas lipídicas desempeñan un papel importante en la homeostasis energética. Estas inclusiones citoplasmáticas almacenan el exceso de energía en forma de triacilgliceroles y en caso de déficit energético, estos se hidrolizarán dando lugar a glicerol y ácidos grasos. El gen *ABHD5* (*α/β-hydrolase domain-containing 5*), también conocido como *CGI-58* (comparative gene identification-58), es miembro de una familia de proteínas que contienen un dominio hidrolasa α/β y se encuentra en el cromosoma 3p21.33. Su producto da lugar a una proteína de 349 aminoácidos con una masa molecular de aproximadamente 39 kD que se ubica en la superficie de las gotas lipídicas del citoplasma [10].

*ABHD5* actúa como coactivador de la proteína triacilglicérido lipasa adiposa (*ATGL*), que es una enzima clave en la regulación e iniciación de la hidrólisis de las gotas lipídicas del citoplasma [10]. Al igual que en *ABHD5*, las mutaciones de *ATGL* producen una enfermedad de almacenamiento de lípidos neutros con acumulación citoplasmática de gotas lipídicas ricas en triglicéridos en múltiples tejidos [11]. A diferencia de Chanarin-Dorfman que cursa con ictiosis, esta se asocia a miopatía.

La ictiosis es una alteración relacionada con defectos en la permeabilidad de la piel. La eritrodermia ictiosiforme está caracterizada por la exposición de escamas blancas delgadas sobre una superficie eritematosa en todo el cuerpo, salvo en pliegues cutáneos de las extremidades, donde predominan las placas de descamación activa [12]. En la piel sana, el espacio entre los estratos córneo y granuloso es ocupado por membranas lamelares lipídicas que aseguran la función estructural y protectora de la piel. Estos lípidos están constituidos principalmente por ceramidas, colesterol y ácidos grasos libres. *ATGL* participa en la síntesis de acilceramida, componente fundamental de la envoltura lipídica de los corneocitos que resulta esencial para mantener la estructura de la epidermis [13]. Por lo tanto, aquellos individuos con una mutación en el gen *ABHD5* presentarán una alteración en la producción de acilceramida, generando una separación de las fases lamelar/no lamelar que da lugar a una piel seca y escamosa.

No existen terapias para el síndrome de Chanarin-Dorfman. El único tratamiento eficaz descrito consiste en restringir de la dieta los ácidos grasos de cadena larga. Además, se ha demostrado que una dieta rica en ácidos grasos de cadena media alivia las lesiones dermatológicas, aunque los niveles de las aminotransferasas se mantienen elevados en el tiempo [14], 15]. La acitretina, un retinoide de segunda generación, también puede mejorar la descamación y el eritema, llegando a reducir de manera significativa la magnitud de estas lesiones [10].

## Puntos de aprendizaje

– La anomalía de Jordans presente en los leucocitos de SP teñidos con May Grünwald-Giemsa es el hallazgo de laboratorio más común de enfermedad de almacenamiento de lípidos neutros.
– La anomalía de Jordans y la ictiosis son rasgos comunes a todos los casos de síndrome de Chanarin-Dorfman.
– La restricción de los ácidos grasos de cadena larga en la dieta es una medida fundamental para controlar las enfermedades de almacenamiento de lípidos neutros.
